# 1-Butyl­quinine tetra­fluoro­borate

**DOI:** 10.1107/S1600536809054907

**Published:** 2010-01-09

**Authors:** Sana Eltaief, Pascal Retailleau, Leo Straver, Béchir Ben Hassine

**Affiliations:** a Laboratoire de Synthèse Organique Asymétrique et Catalyse Homogène (01UR1201), Faculté des Sciences de Monastir, Avenue de l’Environnement, 5019 Monastir, Tunisia; b Institut de Chimie des Substances Naturelles-CNRS, 1 Avenue de la Terrasse, 91198 Gif sur Yvette, France; c Chopinrode 8, 2717 BK Zoetermeer, The Netherlands

## Abstract

In the title salt (2*S*,4*S*,8*R*)-1-butyl-2-[(*R*)-(hydr­oxy)(6-methoxy­quinolin-4-yl)meth­yl]-8-vinyl­quinuclidin-1-ium tetra­fluoro­borate, C_24_H_33_N_2_O_2_^+^·BF_4_^−^, the butyl substituent at the 1-position is in an equatorial conformation with respect to the unsubstituted six-membered ring and the four butyl C atoms are almost coplanar with the ring N and vinyl C atoms (r.m.s. deviation = 0.046 Å).   In the crystal, the cations are linked by O—H⋯N hydrogen bonds. The F atoms of the tetra­fluoro­borate group are disordered over two sets of site with an occupancy raitio of 0.552 (8):0.448 (8).

## Related literature

For the crystal structures of similar 1-butyl­quinine tetra­fluoro­borate derivatives, see: Dijkstra *et al.* (1989 ▶); Samas *et al.* (2005 ▶). For applications of quinine salts, see: Thierry *et al.* (2001 ▶, 2003 ▶). For graph-set notation, see: Bernstein *et al.* (1994 ▶). For a description of the Cambridge Structural Database, see: Allen (2002 ▶).
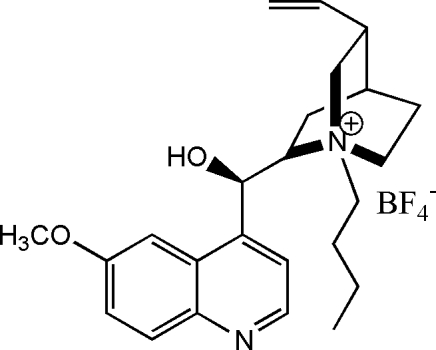


## Experimental

### 

#### Crystal data

C_24_H_33_N_2_O_2_^+^·BF_4_^−^
*M* *_r_* = 468.33
Orthorhombic, 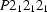

*a* = 8.041 (8) Å
*b* = 12.597 (12) Å
*c* = 22.91 (2) Å
*V* = 2321 (4) Å^3^
*Z* = 4
Mo *K*α radiation
μ = 0.11 mm^−1^
*T* = 120 K
0.60 × 0.20 × 0.15 mm

#### Data collection

Bruker Kappa-APEX DUO diffractometer
Absorption correction: multi-scan (*SADABS*; Bruker, 2008 ▶) *T*_min_ = 0.885, *T*_max_ = 0.982
25415 measured reflections
3968 independent reflections
2957 reflections with *I* > 2σ(*I*)
*R*_int_ = 0.072

#### Refinement

*R*[*F* ^2^ > 2σ(*F* ^2^)] = 0.063
*wR*(*F* ^2^) = 0.156
*S* = 1.06
3968 reflections
329 parameters
H-atom parameters constrained
Δρ_max_ = 0.36 e Å^−3^
Δρ_min_ = −0.38 e Å^−3^

### 

Data collection: *APEX2* (Bruker, 2008 ▶); cell refinement: *SAINT* (Bruker, 2008 ▶); data reduction: *SAINT*; program(s) used to solve structure: *SHELXS97* (Sheldrick, 2008 ▶); program(s) used to refine structure: *SHELXL97* (Sheldrick, 2008 ▶) and *CrystalBuilder* (Welter, 2006 ▶); molecular graphics: *PLATON* (Spek, 2009 ▶) and *Mercury* (Bruno *et al.*, 2004 ▶); software used to prepare material for publication: *SHELXL97* and *publCIF* (Westrip, 2010 ▶).

## Supplementary Material

Crystal structure: contains datablocks global, I. DOI: 10.1107/S1600536809054907/bx2256sup1.cif

Structure factors: contains datablocks I. DOI: 10.1107/S1600536809054907/bx2256Isup2.hkl

Additional supplementary materials:  crystallographic information; 3D view; checkCIF report

## Figures and Tables

**Table 1:** Hydrogen-bond geometry (Å, °)

*D*—H⋯*A*	*D*—H	H⋯*A*	*D*⋯*A*	*D*—H⋯*A*
O1—H1*O*⋯N2^i^	0.84	1.95	2.787 (4)	174

Symmetry code: (i) 
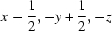
.

**Fig. 1.**
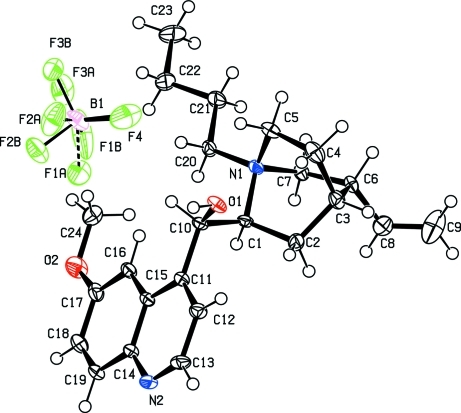
An ORTEP diagram drawing of the title compound, with the atom-numbering scheme and 50% probability displacement ellipsoids.

**Fig. 2.**
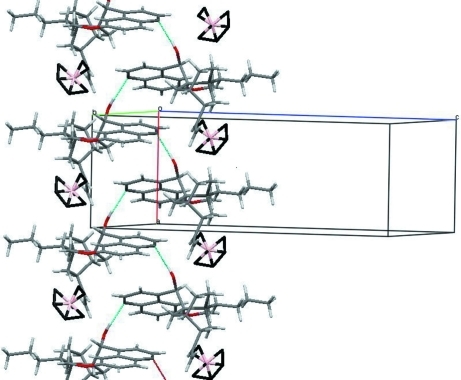
Molecular packing of the title compound as viewed along the a axis.

## supplementary crystallographic information

### Comment

The overall utility of asymmetric catalysts can be compared by examining three main criteria: 1) the variety of reactions that the catalyst can promote, 2) the availability of both enantiomeric antipodes of the catalyst at a reasonable price, and 3) the stability of the catalyst. Alkaloids and quaternary ammonium salt fulfill all of these criteria. They make them one of the most useful catalysts to date. Alkaloids can be transformed to quaternary ammonium salt in one or two steps. Chiral 1-butylquinine cation was reacted with BF_4_^-^ leading to a new salt. The latter could serve as a chiral catalyst for different asymmetric reactions.

The X-ray structure shows that the boron atom presents statistically two types of tetrahedral environments: E1 and E2 with occupancy rates of 55.2% and 44.8%, respectively. The first environment (E1), which consists of F1A, F2A, F3A and F4, is strongly distorted as indicated by the B—F bond lengths varying from 1.324 (5) and 1.468 (5) Å and F—B—F scattering from 99.9 (8) and 123.3 (8)°. The second environment (E2), which consists of F1B, F2B, F3B and F4, is also very distorted as revealed by the B—F distance ranging from 1.298 (5) and 1.437 (5) Å and the F—B—F angles included between 95.5 (7) and 120.3 (8)°.

Regarding the cation, the quinine skeleton displays atomic parameters, which are comparable to those of the forty-two derivatives already deposited at the Cambridge Structural Database (Version 5.30, September 2009 update), Allen, 2002, *Mogul*, Version 1.1.3; Bruno *et al.*, 2004). It commonly adopts the open conformation III described in solution by Dijkstra *et al.*,1989, and in which the butyl-substituted quinuclidine nitrogen, N1, turns away from the quinoline ring and is oriented in the same direction as the methoxy oxygen. The torsion angles, which best characterized the overall shape, C12—C11—C10—O1 and, O1—C10 —C1— C2, are -22.6 (4)° and 45.8 (3)°, respectively. The butyl substituent at N1 is in equatorial conformation with respect to the six-membered ring C3/C7—N1 and the four butyl atoms are almost coplanar with N1—C20/C23 (r.m.s. deviation of 0.046 Å), and parallel to the [001] direction.

In the crystal structure, molecules are mainly linked by intermolecular O—H···N hydrogen bonds into helical chains running along a crystallographic 2_1_ axis at *y*=1/4 position in the *a*-axis direction with graph-set notation C(7) (Bernstein *et al.* (1994). The stability of the chains also benefits from the tilted superimposition of adjacent quinolin moieties with dihedral angle of 36.2 (4)° and shortest centroid distance of 4.162 (5) Å.

### Experimental

Quinine and 1-bromobutane (1.1 equiv) were dissolved in acetonitrile and refluxed overnight. The reaction mixture was concentrated and 1-butylquinine bromide was purified to 95% being washed with ethyl acetate. The desired 1-Butylquinine tetrafluoroborate,[BQ]BF4, was then produced by anion exchange with NaBF_4_ (1.2 equiv) in biphasic CH_2_Cl_2_/H_2_O mixture. The reaction mixture was stirred for a further 24 h. The mixture was then extracted with CH_2_Cl_2_ and the organic phase was dried over MgSO_4_. The solvent evaporation method was used to grow [BQ]BF_4_ crystals in dichloromethane at room temperature. The product is a colorless single-crystal which is air stable (m.p.197- 199 °C).

### Refinement

All H atoms attached to C or O atoms were placed in calculated positions (C—H = 0.95–1.00 Å; O—H = 0.84 Å (hydroxyl)) and allowed to ride on their parent atoms, with *U*_iso_(H) = 1.2*U*_eq_(C_aromatic or vinyl_) or *U*_iso_(H) =1.5*U*_eq_(C_others_, O). 3007 Friedel opposites were merged

### Crystal data

**Table:**

C_24_H_33_N_2_O_2_^+^·BF_4_^−^	*D*_x_ = 1.340 Mg m^−^^3^
*M**_r_* = 468.33	Melting point: 198 K
Orthorhombic, *P*2_1_2_1_2_1_	Mo *K*α radiation, λ = 0.71073 Å
Hall symbol: P 2ac 2ab	Cell parameters from 312 reflections
*a* = 8.041 (8) Å	θ = 2.4–23.5°
*b* = 12.597 (12) Å	µ = 0.11 mm^−^^1^
*c* = 22.91 (2) Å	*T* = 120 K
*V* = 2321 (4) Å^3^	Block, colourless
*Z* = 4	0.60 × 0.20 × 0.15 mm
*F*(000) = 992	

### Data collection

**Table:**

Bruker Kappa-APEX DUO diffractometer	3968 independent reflections
Radiation source: sealed tube	2957 reflections with *I* > 2σ(*I*)
graphite	*R*_int_ = 0.072
Detector resolution: 8.3333 pixels mm^-1^	θ_max_ = 30.5°, θ_min_ = 1.8°
φ and ω scans	*h* = −6→11
Absorption correction: multi-scan (*SADABS*; Bruker, 2008)	*k* = −17→17
*T*_min_ = 0.885, *T*_max_ = 0.982	*l* = −32→32
25415 measured reflections	

### Refinement

**Table:**

Refinement on *F*^2^	Primary atom site location: structure-invariant direct methods
Least-squares matrix: full	Secondary atom site location: difference Fourier map
*R*[*F*^2^ > 2σ(*F*^2^)] = 0.063	Hydrogen site location: difference Fourier map
*wR*(*F*^2^) = 0.156	H-atom parameters constrained
*S* = 1.06	*w* = 1/[σ^2^(*F*_o_^2^) + (0.0531*P*)^2^ + 2.384*P*] where *P* = (*F*_o_^2^ + 2*F*_c_^2^)/3
3968 reflections	(Δ/σ)_max_ < 0.001
329 parameters	Δρ_max_ = 0.36 e Å^−^^3^
0 restraints	Δρ_min_ = −0.38 e Å^−^^3^
0 constraints	

### Special details

**Table:**

Geometry. All e.s.d.'s (except the e.s.d. in the dihedral angle between two l.s. planes) are estimated using the full covariance matrix. The cell e.s.d.'s are taken into account individually in the estimation of e.s.d.'s in distances, angles and torsion angles; correlations between e.s.d.'s in cell parameters are only used when they are defined by crystal symmetry. An approximate (isotropic) treatment of cell e.s.d.'s is used for estimating e.s.d.'s involving l.s. planes.
Refinement. Refinement of *F*^2^ against ALL reflections. The weighted *R*-factor *wR* and goodness of fit *S* are based on *F*^2^, conventional *R*-factors *R* are based on *F*, with *F* set to zero for negative *F*^2^. The threshold expression of *F*^2^ > σ(*F*^2^) is used only for calculating *R*-factors(gt) *etc*. and is not relevant to the choice of reflections for refinement. *R*-factors based on *F*^2^ are statistically about twice as large as those based on *F*, and *R*- factors based on ALL data will be even larger.

### Fractional atomic coordinates and isotropic or equivalent isotropic displacement parameters (Å^2^)

**Table:**

	*x*	*y*	*z*	*U*_iso_*/*U*_eq_	Occ. (<1)
C23	0.6730 (6)	0.0069 (3)	0.38114 (15)	0.0356 (10)	
H23A	0.6159	−0.0618	0.3825	0.053*	
H23B	0.6274	0.0537	0.4113	0.053*	
H23C	0.7922	−0.0037	0.3880	0.053*	
O1	0.4684 (3)	0.02693 (17)	0.06488 (9)	0.0199 (5)	
H1O	0.3809	0.0582	0.0546	0.030*	
O2	0.7800 (4)	0.42741 (19)	0.21386 (10)	0.0322 (7)	
C9	1.1983 (7)	−0.2286 (4)	0.0729 (2)	0.0533 (13)	
H9A	1.1473	−0.2966	0.0725	0.064*	
H9B	1.3072	−0.2197	0.0577	0.064*	
C22	0.6473 (6)	0.0576 (3)	0.32091 (13)	0.0287 (8)	
H22A	0.5272	0.0701	0.3143	0.034*	
H22B	0.7048	0.1271	0.3195	0.034*	
N2	0.6884 (4)	0.3676 (2)	−0.02267 (11)	0.0217 (6)	
C10	0.5780 (4)	0.1006 (2)	0.09150 (12)	0.0158 (6)	
H10	0.5270	0.1271	0.1285	0.019*	
N1	0.7366 (4)	−0.0274 (2)	0.16071 (10)	0.0166 (5)	
C24	0.7511 (5)	0.3433 (3)	0.25445 (13)	0.0256 (8)	
H24A	0.6405	0.3129	0.2477	0.038*	
H24B	0.7573	0.3711	0.2943	0.038*	
H24C	0.8357	0.2881	0.2492	0.038*	
C17	0.7533 (5)	0.4053 (2)	0.15683 (14)	0.0228 (7)	
C16	0.6980 (5)	0.3092 (2)	0.13639 (13)	0.0189 (6)	
H16	0.6765	0.2531	0.1631	0.023*	
C15	0.6724 (4)	0.2928 (2)	0.07579 (12)	0.0162 (6)	
C11	0.6174 (4)	0.1949 (2)	0.05190 (12)	0.0167 (6)	
C1	0.7426 (4)	0.0434 (2)	0.10566 (11)	0.0153 (6)	
H1	0.8281	0.0995	0.1129	0.018*	
C2	0.8059 (5)	−0.0253 (3)	0.05386 (13)	0.0205 (7)	
H2A	0.7304	−0.0168	0.0201	0.025*	
H2B	0.9181	−0.0012	0.0420	0.025*	
C3	0.8128 (5)	−0.1425 (2)	0.07171 (14)	0.0220 (7)	
H3	0.8413	−0.1873	0.0371	0.026*	
C6	0.9446 (4)	−0.1570 (2)	0.11938 (13)	0.0198 (7)	
H6	0.9325	−0.2300	0.1359	0.024*	
C8	1.1178 (5)	−0.1465 (3)	0.09467 (14)	0.0263 (8)	
H8	1.1703	−0.0789	0.0948	0.032*	
C21	0.7152 (5)	−0.0141 (3)	0.27310 (13)	0.0249 (8)	
H21A	0.6534	−0.0821	0.2728	0.030*	
H21B	0.8339	−0.0298	0.2807	0.030*	
C20	0.6969 (5)	0.0408 (2)	0.21390 (12)	0.0200 (7)	
H20A	0.5811	0.0667	0.2102	0.024*	
H20B	0.7708	0.1037	0.2134	0.024*	
C18	0.7836 (5)	0.4904 (3)	0.11797 (16)	0.0280 (8)	
H18	0.8210	0.5568	0.1325	0.034*	
C19	0.7590 (5)	0.4769 (2)	0.05954 (15)	0.0236 (7)	
H19	0.7789	0.5344	0.0337	0.028*	
C14	0.7041 (4)	0.3780 (2)	0.03679 (13)	0.0186 (6)	
C13	0.6389 (5)	0.2759 (3)	−0.04317 (13)	0.0221 (7)	
H13	0.6283	0.2685	−0.0843	0.027*	
C12	0.6002 (4)	0.1872 (3)	−0.00788 (12)	0.0195 (7)	
H12	0.5627	0.1229	−0.0252	0.023*	
C4	0.6443 (5)	−0.1761 (3)	0.09637 (16)	0.0286 (8)	
H4A	0.6440	−0.2535	0.1039	0.034*	
H4B	0.5555	−0.1602	0.0678	0.034*	
C5	0.6115 (5)	−0.1157 (3)	0.15346 (14)	0.0231 (7)	
H5A	0.4977	−0.0857	0.1529	0.028*	
H5B	0.6195	−0.1652	0.1869	0.028*	
C7	0.9085 (4)	−0.0766 (2)	0.16817 (13)	0.0183 (6)	
H7A	0.9148	−0.1127	0.2065	0.022*	
H7B	0.9938	−0.0200	0.1675	0.022*	
B1	0.2102 (6)	0.1749 (3)	0.21354 (18)	0.0298 (10)	
F4	0.2425 (5)	0.0825 (2)	0.18461 (12)	0.0708 (11)	
F1A	0.3521 (8)	0.2072 (5)	0.2369 (4)	0.078 (3)	0.552 (8)
F2A	0.1287 (7)	0.2640 (4)	0.18703 (19)	0.0410 (15)	0.552 (8)
F3A	0.0935 (13)	0.1528 (5)	0.2613 (3)	0.085 (3)	0.552 (8)
F1B	0.3276 (11)	0.2428 (5)	0.1845 (4)	0.066 (3)	0.448 (8)
F2B	0.0631 (9)	0.1900 (7)	0.1900 (3)	0.056 (3)	0.448 (8)
F3B	0.2247 (13)	0.1795 (5)	0.2699 (3)	0.049 (2)	0.448 (8)

### Atomic displacement parameters (Å^2^)

**Table:**

	*U*^11^	*U*^22^	*U*^33^	*U*^12^	*U*^13^	*U*^23^
C23	0.045 (3)	0.045 (2)	0.0171 (15)	−0.006 (2)	0.0005 (16)	0.0078 (14)
O1	0.0225 (14)	0.0132 (10)	0.0241 (11)	−0.0040 (10)	−0.0072 (10)	0.0040 (8)
O2	0.0489 (19)	0.0218 (12)	0.0258 (12)	−0.0070 (12)	−0.0034 (12)	−0.0064 (9)
C9	0.034 (3)	0.069 (3)	0.058 (3)	0.004 (3)	0.003 (2)	−0.028 (3)
C22	0.039 (2)	0.0311 (18)	0.0162 (14)	0.0057 (17)	0.0041 (14)	0.0043 (12)
N2	0.0248 (17)	0.0199 (13)	0.0202 (12)	0.0009 (12)	0.0044 (11)	0.0079 (10)
C10	0.0197 (18)	0.0124 (13)	0.0151 (12)	−0.0009 (12)	0.0003 (12)	0.0043 (10)
N1	0.0216 (16)	0.0138 (11)	0.0144 (11)	0.0030 (11)	0.0001 (10)	0.0030 (9)
C24	0.027 (2)	0.0300 (18)	0.0200 (14)	−0.0028 (15)	−0.0006 (14)	−0.0055 (12)
C17	0.029 (2)	0.0153 (14)	0.0237 (15)	0.0020 (14)	0.0013 (14)	−0.0024 (11)
C16	0.0259 (19)	0.0134 (13)	0.0175 (13)	0.0022 (13)	−0.0005 (12)	0.0025 (10)
C15	0.0202 (18)	0.0123 (13)	0.0159 (13)	0.0011 (12)	0.0007 (12)	0.0029 (10)
C11	0.0194 (18)	0.0142 (14)	0.0166 (13)	0.0017 (13)	0.0007 (12)	0.0032 (10)
C1	0.0217 (18)	0.0132 (13)	0.0111 (11)	−0.0020 (12)	0.0001 (11)	0.0037 (9)
C2	0.0266 (19)	0.0202 (14)	0.0148 (13)	0.0042 (14)	0.0012 (12)	−0.0007 (11)
C3	0.027 (2)	0.0144 (14)	0.0244 (15)	−0.0011 (14)	−0.0011 (14)	−0.0042 (11)
C6	0.0255 (19)	0.0110 (13)	0.0228 (14)	0.0019 (12)	0.0020 (14)	−0.0001 (11)
C8	0.028 (2)	0.0289 (17)	0.0221 (15)	−0.0003 (16)	−0.0015 (14)	−0.0016 (13)
C21	0.035 (2)	0.0229 (16)	0.0172 (14)	0.0070 (15)	−0.0005 (14)	0.0069 (11)
C20	0.0296 (19)	0.0160 (14)	0.0144 (12)	0.0069 (13)	0.0020 (13)	0.0019 (10)
C18	0.037 (2)	0.0119 (14)	0.0348 (18)	−0.0024 (14)	0.0053 (17)	−0.0017 (12)
C19	0.028 (2)	0.0117 (14)	0.0311 (16)	−0.0004 (14)	0.0078 (15)	0.0041 (11)
C14	0.0201 (18)	0.0139 (13)	0.0218 (14)	0.0017 (13)	0.0041 (12)	0.0053 (10)
C13	0.025 (2)	0.0260 (16)	0.0153 (13)	−0.0010 (14)	0.0009 (13)	0.0078 (12)
C12	0.0227 (19)	0.0191 (14)	0.0167 (13)	−0.0005 (13)	−0.0007 (12)	0.0022 (11)
C4	0.031 (2)	0.0164 (15)	0.0379 (19)	−0.0060 (14)	−0.0064 (16)	−0.0028 (13)
C5	0.027 (2)	0.0158 (14)	0.0269 (15)	−0.0038 (14)	−0.0017 (14)	0.0062 (12)
C7	0.0183 (18)	0.0165 (14)	0.0201 (14)	0.0031 (13)	−0.0019 (12)	0.0018 (11)
B1	0.041 (3)	0.0181 (17)	0.0301 (19)	−0.0058 (18)	−0.0086 (19)	0.0032 (14)
F4	0.108 (3)	0.0438 (15)	0.0606 (17)	−0.0434 (18)	0.0418 (18)	−0.0335 (13)
F1A	0.046 (4)	0.046 (3)	0.142 (8)	0.018 (3)	−0.054 (5)	−0.054 (5)
F2A	0.059 (4)	0.025 (2)	0.039 (2)	0.007 (2)	−0.009 (2)	0.0031 (17)
F3A	0.120 (8)	0.058 (4)	0.077 (5)	0.052 (5)	0.068 (5)	0.036 (3)
F1B	0.069 (6)	0.022 (3)	0.106 (7)	−0.026 (3)	0.051 (5)	−0.020 (3)
F2B	0.037 (4)	0.084 (7)	0.046 (3)	0.027 (4)	−0.006 (3)	−0.008 (4)
F3B	0.077 (7)	0.043 (3)	0.026 (3)	0.014 (4)	−0.013 (3)	−0.015 (2)

### Geometric parameters (Å, °)

**Table:**

C23—C22	1.535 (5)	C1—C2	1.555 (4)
C23—H23A	0.9800	C1—H1	1.0000
C23—H23B	0.9800	C2—C3	1.533 (5)
C23—H23C	0.9800	C2—H2A	0.9900
O1—C10	1.417 (4)	C2—H2B	0.9900
O1—H1O	0.8400	C3—C4	1.528 (6)
O2—C17	1.353 (4)	C3—C6	1.533 (5)
O2—C24	1.429 (4)	C3—H3	1.0000
C9—C8	1.317 (6)	C6—C8	1.509 (5)
C9—H9A	0.9500	C6—C7	1.536 (4)
C9—H9B	0.9500	C6—H6	1.0000
C22—C21	1.522 (5)	C8—H8	0.9500
C22—H22A	0.9900	C21—C20	1.530 (4)
C22—H22B	0.9900	C21—H21A	0.9900
N2—C13	1.310 (5)	C21—H21B	0.9900
N2—C14	1.374 (4)	C20—H20A	0.9900
C10—C11	1.528 (4)	C20—H20B	0.9900
C10—C1	1.541 (5)	C18—C19	1.364 (5)
C10—H10	1.0000	C18—H18	0.9500
N1—C5	1.509 (4)	C19—C14	1.421 (5)
N1—C7	1.524 (4)	C19—H19	0.9500
N1—C20	1.525 (4)	C13—C12	1.414 (4)
N1—C1	1.545 (4)	C13—H13	0.9500
C24—H24A	0.9800	C12—H12	0.9500
C24—H24B	0.9800	C4—C5	1.536 (5)
C24—H24C	0.9800	C4—H4A	0.9900
C17—C16	1.372 (4)	C4—H4B	0.9900
C17—C18	1.415 (5)	C5—H5A	0.9900
C16—C15	1.419 (4)	C5—H5B	0.9900
C16—H16	0.9500	C7—H7A	0.9900
C15—C14	1.419 (4)	C7—H7B	0.9900
C15—C11	1.421 (4)	B1—F4	1.364 (5)
C11—C12	1.380 (4)		
			
C17—O2—C24	116.7 (3)	C8—C6—C7	112.9 (3)
C8—C9—H9A	120.0	C3—C6—C7	108.0 (3)
C8—C9—H9B	120.0	C8—C6—H6	108.2
H9A—C9—H9B	120.0	C3—C6—H6	108.2
C21—C22—C23	110.6 (3)	C7—C6—H6	108.2
C21—C22—H22A	109.5	C9—C8—C6	121.8 (4)
C23—C22—H22A	109.5	C9—C8—H8	119.1
C21—C22—H22B	109.5	C6—C8—H8	119.1
C23—C22—H22B	109.5	C22—C21—C20	109.6 (3)
H22A—C22—H22B	108.1	C22—C21—H21A	109.8
C13—N2—C14	117.8 (3)	C20—C21—H21A	109.8
O1—C10—C11	112.5 (2)	C22—C21—H21B	109.8
O1—C10—C1	108.6 (2)	C20—C21—H21B	109.8
C11—C10—C1	108.1 (3)	H21A—C21—H21B	108.2
O1—C10—H10	109.2	N1—C20—C21	115.7 (2)
C11—C10—H10	109.2	N1—C20—H20A	108.4
C1—C10—H10	109.2	C21—C20—H20A	108.4
C5—N1—C7	108.5 (2)	N1—C20—H20B	108.4
C5—N1—C20	111.3 (3)	C21—C20—H20B	108.4
C7—N1—C20	109.2 (2)	H20A—C20—H20B	107.4
C5—N1—C1	110.9 (2)	C19—C18—C17	119.9 (3)
C7—N1—C1	107.4 (2)	C19—C18—H18	120.1
C20—N1—C1	109.5 (2)	C17—C18—H18	120.1
O2—C17—C16	124.3 (3)	C18—C19—C14	121.0 (3)
O2—C17—C18	115.1 (3)	C18—C19—H19	119.5
C16—C17—C18	120.6 (3)	C14—C19—H19	119.5
C17—C16—C15	120.6 (3)	N2—C14—C15	122.4 (3)
C17—C16—H16	119.7	N2—C14—C19	118.4 (3)
C15—C16—H16	119.7	C15—C14—C19	119.2 (3)
C16—C15—C14	118.7 (3)	N2—C13—C12	124.0 (3)
C16—C15—C11	123.3 (3)	N2—C13—H13	118.0
C14—C15—C11	118.0 (3)	C12—C13—H13	118.0
C12—C11—C15	118.3 (3)	C11—C12—C13	119.4 (3)
C12—C11—C10	120.9 (3)	C11—C12—H12	120.3
C15—C11—C10	120.7 (3)	C13—C12—H12	120.3
C10—C1—N1	114.5 (3)	C3—C4—C5	109.3 (3)
C10—C1—C2	112.4 (2)	C3—C4—H4A	109.8
N1—C1—C2	108.2 (2)	C5—C4—H4A	109.8
C10—C1—H1	107.1	C3—C4—H4B	109.8
N1—C1—H1	107.1	C5—C4—H4B	109.8
C2—C1—H1	107.1	H4A—C4—H4B	108.3
C3—C2—C1	110.2 (3)	N1—C5—C4	110.2 (3)
C3—C2—H2A	109.6	N1—C5—H5A	109.6
C1—C2—H2A	109.6	C4—C5—H5A	109.6
C3—C2—H2B	109.6	N1—C5—H5B	109.6
C1—C2—H2B	109.6	C4—C5—H5B	109.6
H2A—C2—H2B	108.1	H5A—C5—H5B	108.1
C4—C3—C6	108.5 (3)	N1—C7—C6	111.0 (3)
C4—C3—C2	109.4 (3)	N1—C7—H7A	109.4
C6—C3—C2	109.3 (3)	C6—C7—H7A	109.4
C4—C3—H3	109.9	N1—C7—H7B	109.4
C6—C3—H3	109.9	C6—C7—H7B	109.4
C2—C3—H3	109.9	H7A—C7—H7B	108.0
C8—C6—C3	111.1 (3)		
			
C24—O2—C17—C16	1.4 (6)	C7—C6—C8—C9	150.3 (4)
C24—O2—C17—C18	−179.9 (3)	C23—C22—C21—C20	−176.9 (3)
O2—C17—C16—C15	179.7 (4)	C5—N1—C20—C21	66.9 (4)
C18—C17—C16—C15	1.0 (6)	C7—N1—C20—C21	−52.9 (4)
C17—C16—C15—C14	−0.4 (5)	C1—N1—C20—C21	−170.2 (3)
C17—C16—C15—C11	179.2 (3)	C22—C21—C20—N1	−171.6 (3)
C16—C15—C11—C12	−178.6 (3)	O2—C17—C18—C19	−179.4 (4)
C14—C15—C11—C12	1.0 (5)	C16—C17—C18—C19	−0.5 (6)
C16—C15—C11—C10	1.5 (5)	C17—C18—C19—C14	−0.4 (6)
C14—C15—C11—C10	−178.9 (3)	C13—N2—C14—C15	1.4 (5)
O1—C10—C11—C12	−22.6 (4)	C13—N2—C14—C19	179.5 (3)
C1—C10—C11—C12	97.2 (4)	C16—C15—C14—N2	177.6 (3)
O1—C10—C11—C15	157.3 (3)	C11—C15—C14—N2	−2.1 (5)
C1—C10—C11—C15	−82.9 (4)	C16—C15—C14—C19	−0.5 (5)
O1—C10—C1—N1	−78.1 (3)	C11—C15—C14—C19	179.9 (3)
C11—C10—C1—N1	159.6 (2)	C18—C19—C14—N2	−177.2 (4)
O1—C10—C1—C2	45.8 (3)	C18—C19—C14—C15	0.9 (6)
C11—C10—C1—C2	−76.4 (3)	C14—N2—C13—C12	0.2 (6)
C5—N1—C1—C10	62.0 (3)	C15—C11—C12—C13	0.5 (5)
C7—N1—C1—C10	−179.7 (2)	C10—C11—C12—C13	−179.6 (3)
C20—N1—C1—C10	−61.2 (3)	N2—C13—C12—C11	−1.2 (6)
C5—N1—C1—C2	−64.1 (3)	C6—C3—C4—C5	53.4 (3)
C7—N1—C1—C2	54.2 (3)	C2—C3—C4—C5	−65.7 (3)
C20—N1—C1—C2	172.7 (3)	C7—N1—C5—C4	−65.4 (3)
C10—C1—C2—C3	−117.9 (3)	C20—N1—C5—C4	174.4 (3)
N1—C1—C2—C3	9.5 (4)	C1—N1—C5—C4	52.2 (3)
C1—C2—C3—C4	53.0 (4)	C3—C4—C5—N1	11.5 (4)
C1—C2—C3—C6	−65.7 (4)	C5—N1—C7—C6	51.3 (3)
C4—C3—C6—C8	168.6 (3)	C20—N1—C7—C6	172.8 (2)
C2—C3—C6—C8	−72.2 (3)	C1—N1—C7—C6	−68.5 (3)
C4—C3—C6—C7	−67.0 (3)	C8—C6—C7—N1	136.1 (3)
C2—C3—C6—C7	52.2 (4)	C3—C6—C7—N1	12.8 (3)
C3—C6—C8—C9	−88.1 (4)		

### Hydrogen-bond geometry (Å, °)

**Table:**

*D*—H···*A*	*D*—H	H···*A*	*D*···*A*	*D*—H···*A*
O1—H1O···N2^i^	0.84	1.95	2.787 (4)	174

Symmetry codes: (i) *x*−1/2, −*y*+1/2, −*z*.
